# Role of Leptin in the Digestive System

**DOI:** 10.3389/fphar.2021.660040

**Published:** 2021-04-12

**Authors:** Min-Hyun Kim, Hyeyoung Kim

**Affiliations:** ^1^ Department of Molecular and Integrative Physiology, University of Michigan Medical School, Ann Arbor, MI, United States; ^2^ Department of Food and Nutrition, College of Human Ecology, Yonsei University, Seoul, Korea

**Keywords:** leptin, digestive system, signal transduction, cytoprotection, immune system

## Abstract

Leptin is a pluripotent peptide hormone produced mainly by adipocytes, as well as by other tissues such as the stomach. Leptin primarily acts on the central nervous system, particularly the hypothalamus, where this hormone regulates energy homeostasis and neuroendocrine function. Owing to this, disruption of leptin signaling has been linked with numerous pathological conditions. Recent studies have also highlighted the diverse roles of leptin in the digestive system including immune regulation, cell proliferation, tissue healing, and glucose metabolism. Of note, leptin acts differently under physiological and pathological conditions. Here, we review the current knowledge on the functions of leptin and its downstream signaling in the gastrointestinal tract and accessory digestive organs, with an emphasis on its physiological and pathological implications. We also discuss the current therapeutic uses of recombinant leptin, as well as its limitations.

## Introduction

Leptin, a 16 kDa hormone encoded by the *ob* gene, is mainly produced and secreted by adipocytes (Friedman, 2019). In general, circulating leptin levels reflect body fat mass (Francisco et al., 2018). Leptin is also produced by other tissues, including the stomach, skeletal muscles, pituitary gland, and mammary gland (Reid et al., 2018; Inagaki-Ohara, 2019; Zieba et al., 2020). Leptin was discovered as a central regulator of systemic energy homeostasis (Ahima et al., 1996). Under normal conditions, elevated circulating leptin levels suppress feeding behavior while elevating energy expenditure by modulating multiple neuroendocrine axes (Pandit et al., 2017). On the other hand, decreased circulating leptin levels stimulate feeding behavior while decreasing energy utilization. Adequate levels of functional leptin are required for maintaining several physiological functions, including reproduction, tissue remodeling, growth and development, and the immune system (Ramos-Lobo and Donato Jr, 2017; Maurya et al., 2018; Monteiro et al., 2019; Sengupta et al., 2019).

Leptin function is mediated by its binding with the leptin receptor (LepR), which has multiple isoforms due to alternative splicing of LepR mRNA (Tartaglia et al., 1995; Wauman et al., 2017). Among these, the longest isoform, LepRb, mediates most of the known physiologic functions of leptin (Burguera et al., 2000; Wada et al., 2014) Activated LepRb regulates several downstream signaling pathways. These include the Janus kinase 2 (JAK2)/signal transducer and activator of transcription 3 (STAT3) and STAT5, extracellular-signal-regulated kinase (ERK), and phosphoinositide-3 kinase (PI3K) pathways (Liu et al., 2018). Leptin-mediated STAT3 activation results in feedback inhibition by increasing the expression of suppressor of cytokine signaling 3 (SOCS3) and protein tyrosine phosphatase 1 B (PTP1B), which decreases leptin sensitivity (Zhang et al., 2015). The short isoforms of LepR lack the intracellular domain required for signal transduction (Nunziata et al., 2019). These isoforms seem to be involved in the transport and degradation of leptin in tissues, thereby buffers leptin levels in tissues (Schaab et al., 2015). However, their functional activity is believed to be insignificant compared to the LepRb.

Most of the leptin functions have been attributed to its action in the brain, particularly in the hypothalamus, where LepRb is highly expressed (Van Swieten et al., 2014). In addition to that in the brain, LepRb expression has been widely detected in many other tissues, including immune cells and digestive organs (Wada et al., 2014). In the digestive system, leptin has been shown to play several roles, including regulation of immune responses, supporting cell growth and tissue repair, and regulation of glucose and lipid metabolism (Reidy et al., 2000; Marra, 2007; Fernández-Riejos et al., 2010). Leptin appears to play a complex role in these tissues, acting as either beneficial or deleterious depending on the physiological context. Notably, leptin is a member of the helical cytokine family along with IL-6 and IL-12, and LepR belongs to the group of class I cytokine receptors (Conde et al., 2014; Francisco et al., 2018). Therefore, leptin augments immune responses as a proinflammatory cytokine, which may lead to harmful consequences in inflammatory diseases (La Cava, 2017). Here, we review the current knowledge on the functions of leptin in the digestive system, including the gastrointestinal tract (stomach and intestine) and accessory digestive organs (liver and pancreas), with an emphasis on its physiological and pathological implications. We also discuss the possible therapeutic uses of leptin (to boost leptin signaling) or leptin antagonists (to suppress leptin signaling) in the digestive organs, and its limitations. We searched multiple databases including Pubmed, Scopus, and Web of Science (all dates) to identify relevant studies (total 183 articles identified). We only included peer-reviewed articles with a robust experimental design (28 articles excluded).

## Stomach

Leptin expression is not restricted to adipose tissues; a significant amount of leptin is also produced by the stomach (Kasacka et al., 2019). Bado et al. initially discovered that leptin is expressed in rat stomach epithelium (Bado et al., 1998), and other researchers later observed leptin expression in the human stomach as well (Breidert et al., 1999; Sobhani et al., 2000). The release of gastric leptin is stimulated by food consumption, food digestion, and hormones such as cholecystokinin (CCK), gastrin, or secretin (Camilleri and obesity, 2019; Goyal et al., 2019). Upon secretion, leptin displays resistance to proteolysis in the gastric juice, maintaining its functional structure (Buyse et al., 2001). In the stomach, leptin interacts with CCK to increase vagal afferent activities. This action controls the gastric emptying of ingested food, contributing to satiety (Goyal et al., 2019). Full-length LepRb and four short isoforms are expressed in the membranes of fundic and antral gastric cells (Mix et al., 2000; Sobhani et al., 2000). Despite of the receptor expression, however, a direct action of leptin on gastric epithelial cell function is insufficiently understood. Instead, autonomic activity exerted by leptin’s action in the central nervous system (CNS) seems to be more important mechanism to govern physiology of the stomach (Tanida et al., 2019).

Studies using leptin-deficient *ob/ob* mice suggest that normal leptin signaling is critical for healing of gastric injury. In acetic acid-mediated experimental gastric ulcer model, *ob/ob* mice display impaired ulcer healing compared to that in wild-type mice due to the reduced expression of vascular endothelial growth factor (VEGF) and impairment of angiogenesis (Tanigawa et al., 2010). In the same model, restoration of leptin levels in *ob/ob* mice via leptin injection reverses the impaired ulcer healing owing to increased VEGF expression in gastric ulcerous tissue (Tanigawa et al., 2010). In ischemia reperfusion- and ethanol-induced gastric ulceration models, rats administered leptin [10 μg/kg body weight (BW)] showed significantly attenuated gastric lesions (Brzozowski et al., 2001). Notably, the protection conferred by leptin is as effective as that by CCK-8, a potent gastric protector (Mirza et al., 2018). Consistently, in rats with either indomethacin-induced or stress-induced gastric ulcer, leptin treatment (10 μg/kg BW, 6 h) significantly decreased the gastric ulcer index and neutrophil infiltration (Motawi et al., 2008; Khalefa et al., 2010). Several studies using rodent models highlight possible mechanisms. In rats with ulcers, leptin treatment (10 μg/kg BW) supports ulcer healing via production of nitric oxide (NO) and histamine, suppression of oxidative stress, and enhanced expression of transforming growth factor-α (TGF-α), a critical growth promoting factor for the gastric mucosa (Konturek et al., 2001; Erkasap et al., 2003; Motawi et al., 2008).

**FIGURE 1:**
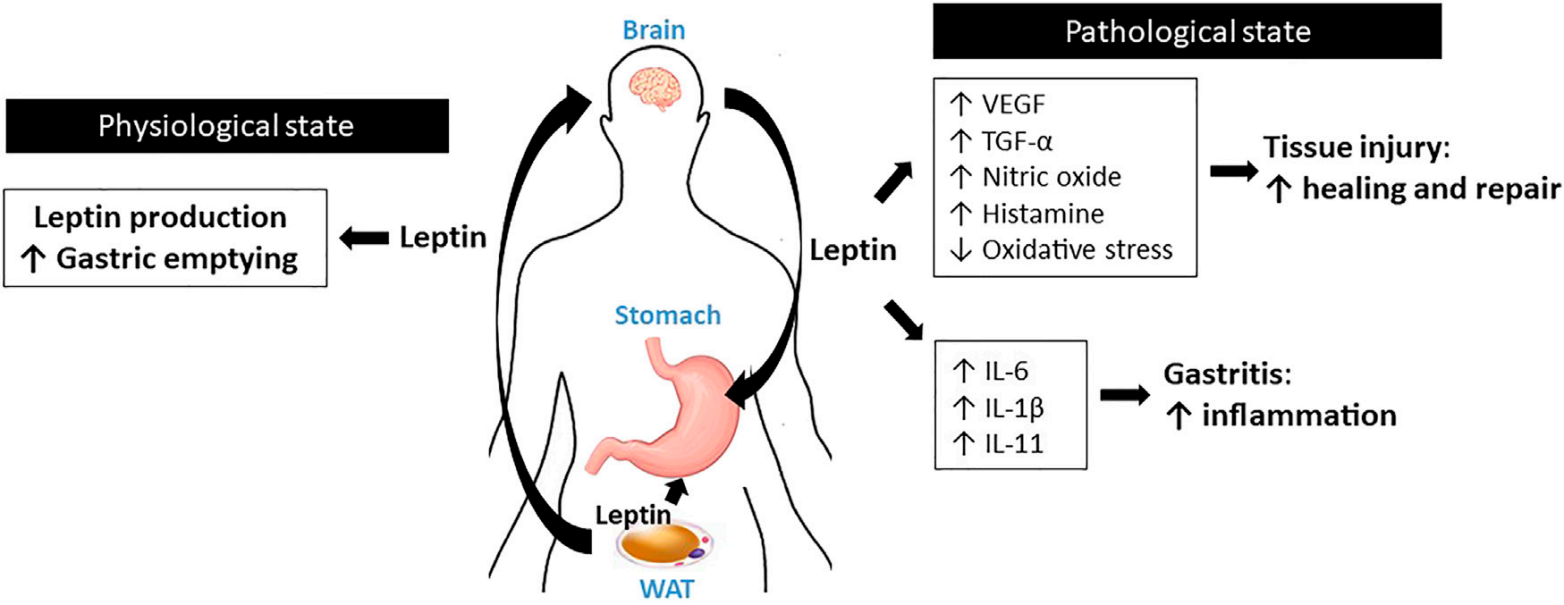
Schematic diagram for the roles of leptin in the stomach.

Nevertheless, unlike its protective effects against gastric injury, leptin appears to accelerate immune responses during gastric inflammation by synergistically interacting with a number of proinflammatory cytokines. Inagaki-Ohara et al. reported that feeding a high-fat diet promotes gastric intestinal metaplasia and atrophic gastritis via the activation of leptin signaling in C57BL6 wild-type mice (Inagaki-Ohara et al., 2016). On the contrary, *ob/ob* mice and LepR-deficient *db/db* mice demonstrate markedly suppressed gastric intestinal metaplasia and expression of proinflammatory cytokines, such as interleukin-6 (IL-6) and IL-11, under the same conditions, indicating that systemic leptin signaling is required to mediate proinflammatory responses (Inagaki-Ohara et al., 2016). Leptin has been implicated in several experimental models of *Helicobacter pylori* infection, which is the major cause of chronic gastritis and peptic ulcer diseases (Kang and Kim, 2017). Several clinical studies have demonstrated that mucosal leptin levels are significantly elevated in *H. pylori*-infected patients compared to those in uninfected individuals (Breidert et al., 1999; Azuma et al., 2001; Nishi et al., 2005; Roper et al., 2008; Romo-González et al., 2017). Using biopsy samples of *H. pylori-*infected patients*,* Nishi et al. showed that gastric leptin levels are positively correlated with the gastric levels of proinflammatory cytokines, including IL-1β and IL-6, indicating that leptin may modulate inflammatory responses during *H. pylori infection* (Nishi et al., 2005) (Figure 1).

## Intestine

Leptin has been shown to modulate intestinal functions, mostly via its action on CNS as well as its regulation of vagal afferent sensitivity to intestinal signals (Huang et al., 2021). Significant expression of LepRb, the longest functional isoform, has been detected in Caco-2 and rat intestinal mucosal cells (Buyse et al., 2001), as well as in the brush border, basolateral membrane, and enterocytes of the human intestine (Barrenetxe et al., 2002). In the intestine, leptin has been shown to control the absorption of macronutrients. Although leptin decreases carbohydrate absorption during the pre-prandial state, leptin increases carbohydrate absorption during the postprandial state via increased expression of carbohydrate transporters, glucose transporter 2 (GLUT-2), GLUT-5, and sodium-glucose cotransporter-1 (SGLT-1) (Pearson et al., 2001; Alavi et al., 2002; Sakar et al., 2009). Leptin-mediated upregulation of GLUT2/5 couples with the activation of protein kinase C subunit βII (PKCβII) and 5′AMP-activated protein kinase subunit α (AMPKα), which are the central players in glucose uptake (Sakar et al., 2009). Leptin also increases protein absorption via activation of the proton-dependent di- and tri-peptide transporter PepT1 (Buyse et al., 2001). In contrast, following food ingestion, leptin reduces lipid release into the circulation by suppressing apolipoprotein (Apo) A-IV (Doi et al., 2001), B-100, and B-48 (Stan et al., 2001).

Leptin is required for gut development and maintenance, as it functions as a growth factor for intestinal cells (Martin and Devkota, 2018). Indeed, *ob/ob* and *db/db* mice display diminished intestinal barrier function and increased intestinal permeability relative to wild-type littermates (Thaiss et al., 2018). (Alavi et al., 2020) reported that systemic leptin administration [6.25–43.75 (mu)g/kg/d; total 14 days] to rats enhances mucosal mass, thereby supporting normal gut physiology (Alavi et al., 2002). In C57BL/6 wild-type mice, acute leptin treatment (10 mg/kg BW; 15 h) promotes colonic epithelial cell proliferation. This increased proliferation is mediated by activation of the p42/44 mitogen-activated protein kinase (MAPK) pathway (Hardwick et al., 2001). Several lines of evidence indicate that leptin improves tissue injury healing and mucosal defense mechanisms. Leptin stimulates mucin secretion by activating the PKC- and PI3K pathways in human colonic goblet-like HT29-MTX cells (Plaisancie et al., 2006). In rats with anastomotic leaks, leptin administration (1 mg/kg BW, i. p.) leads to increased vascular tissue proliferation, collagen tissue proliferation, and mononuclear leukocyte infiltration, which accelerates the healing of colonic damage (Tasdelen et al., 2004). Similarly, in rats with ischemia-reperfusion injury, leptin treatment (100 μg/kg BW) significantly reduces tissue injury (Hacioglu et al., 2005). A study using rat arteries suggested that the tissue healing effect of leptin may be attributed to its stimulation of nitric oxide production, which mediates vasodilation, which in turn assists wound healing (Kimura et al., 2000).

**FIGURE 2:**
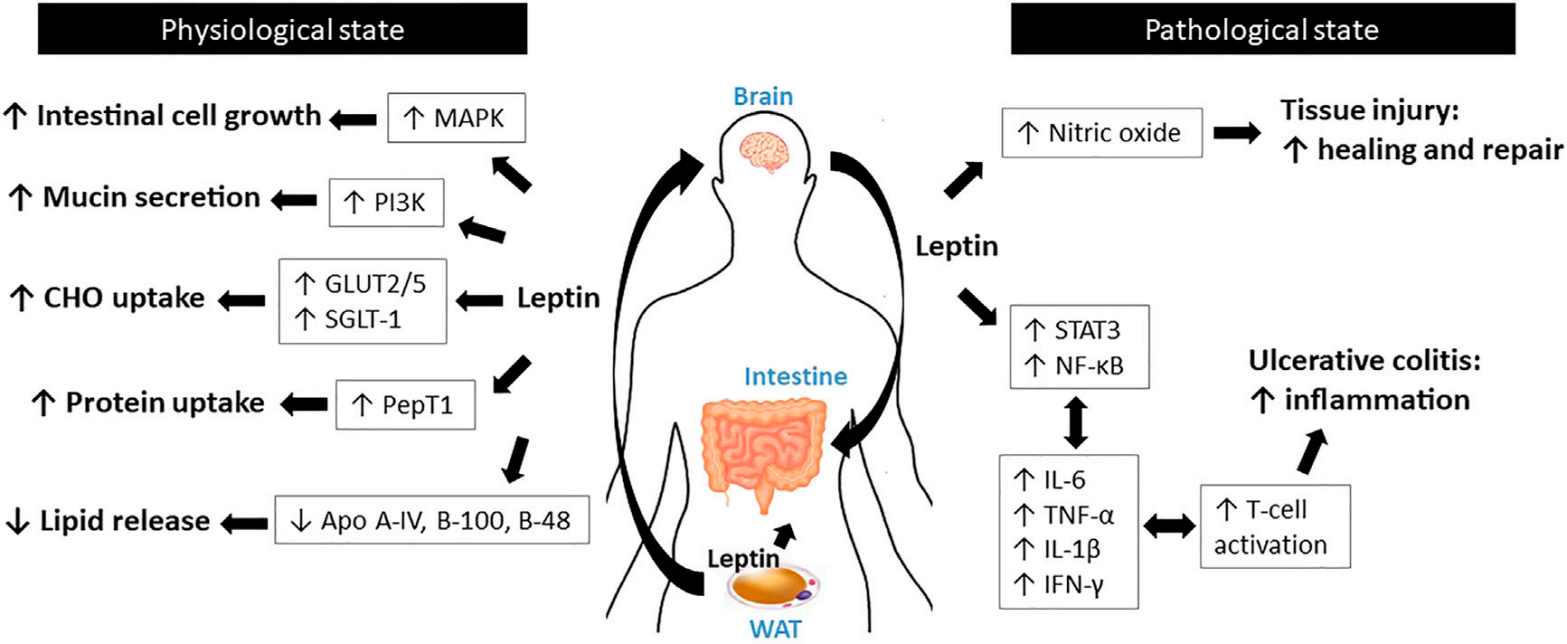
Schematic diagram for the roles of leptin in the intestine.

Inflammatory bowel disease (IBD) comprises Crohn’s disease (CD) and ulcerative colitis (UC) (Jarmakiewicz-Czaja et al., 2020). The common features of IBD include body weight loss, anorexia, and higher energy expenditure (Sairenji et al., 2017). As leptin is a central hormone involved in energy homeostasis and neuroendocrine function (Francisco et al., 2018), its involvement in IBD has been examined by many researchers. In both experimental *in vivo* IBD models and in human patients with IBD, serum leptin levels are elevated (Tuzun et al., 2004; Biesiada et al., 2012). In addition, LepR expression is also elevated in the mesenteric adipose tissue of patients with CD and UC (Barbier et al., 2003; Siegmund, 2012; Zulian et al., 2012). These data indicate that systemic leptin signaling is activated in IBD. Multiple researches show that the increased leptin signaling activates immune responses leading to IBD aggravation, resulting in deleterious effects. It should be noted that *ob/ob* and *db/db* mice are more resistant to experimental colitis models than the wild-type littermates (Siegmund et al., 2002). In dextran sulfate sodium (DSS)- and trinitrobenzene sulfonic acid (TNBS)-induced colitis models, *ob/ob* mice produce significantly low levels of proinflammatory cytokines, such as interferon-γ (IFN-γ), tumor necrosis factor-α (TNF-α), IL-1β, and IL-6, which coincide with reduced STAT3 phosphorylation and cyclooxygenase-2 (COX-2) expression (Siegmund et al., 2002). However, administration of leptin to *ob/ob* mice is able to revert their resistance to colitis. In another study, researchers isolated T-cells from either *db/db* mice or wild-type mice and introduced them into immune-deficient *scid* mice (Siegmund et al., 2004). Strikingly, T-cells isolated from *db/db* mice delayed the development of colitis, indicating that the leptin/LepR axis is required for colitis progression by activating the T lymphocytes (Siegmund et al., 2004). Sitaraman et al., 2004 reported that inflamed colonic epithelial cells express and release leptin into the intestinal lumen (Sitaraman et al., 2004). Luminal leptin then activates nuclear factor kappa B (NF-κB), a potent pro-inflammation stimulator, which results in epithelial wall damage and neutrophil infiltration. Not only proinflammatory responses, leptin may mediates anorexia and body weight loss in IBD. In rats with TNBS-induced colitis, the severity of colitis is associated with circulating leptin levels as well as with anorexia and body weight loss (Barbier et al., 1998). In mice with DSS-induced colitis, delayed puberty is observed in proportion with the changes in serum leptin concentration, food intake, and body weight (Deboer and Li, 2011) (Figure 2).

## Liver

Unlike the other tissues discussed above, where the longest form, LepRb, is highly expressed, the liver appears to only express short forms of LepRs (Zhao et al., 2000; Otte et al., 2004). The absence of functional LepRb indicates that liver is unlikely a direct target of leptin. However, leptin has been shown to interact with various hepatic metabolic pathways, such as glucose and lipid metabolism, possibly via its function in the CNS (da Silva et al., 2020). Leptin controls glucose homeostasis by suppressing the hepatic *de novo* gluconeogenesis and lipogenesis and increasing hepatic triglyceride secretion (Denroche et al., 2012; Hackl et al., 2019) as well as modulating insulin activity through the stimulation insulin receptor substrate-1 (IRS-1)-associated PI3K activity (Cohen et al., 1996). Leptin prevents hepatic lipotoxicity by confining the storage of triglycerides to adipocytes (Unger et al., 1999; Engin and Engin, 2017).

Aberrant leptin signaling has been implicated in non-alcoholic fatty liver diseases (NAFLD) such as hepatic steatosis, hepatitis, and fibrosis. Spontaneous liver steatosis develops in *ob/ob* mice and in LepR-mutated *fa/fa* Zucker rats (Laiglesia et al., 2018; Martinelli et al., 2020). Mechanistically, *ob/ob* mice exhibit significantly higher hepatic peroxisome proliferator-activated receptor γ (PPARγ) activation, which elevates hepatic triglyceride levels by targeting the lipid-associated genes such as *Fsp27* (Matsusue et al., 2008). In the liver of *db/db* mice, level of miR-30c-5p is markedly reduced. Since miR-30c-5p is a direct suppressor of fatty acid synthase (FASN) expression, the reduction of miR-30c-5p upregulates fatty acid synthase (FASN), which elevates fatty acid biosynthesis (Fan et al., 2017). Multiple studies conducted in rodents and humans have shown that leptin treatment may ameliorate hepatic fat accumulation, suggesting its anti-steatotic effect (Paz-Filho et al., 2015; Rodríguez et al., 2015; Boutari and Mantzoros, 2020). Leptin also controls hepatic sympathetic nerve activity via activation of PI3K and AMPK signaling, which leads to improvement in the fatty liver disease (Miyamoto et al., 2012; Tanida et al., 2015).

**FIGURE 3:**
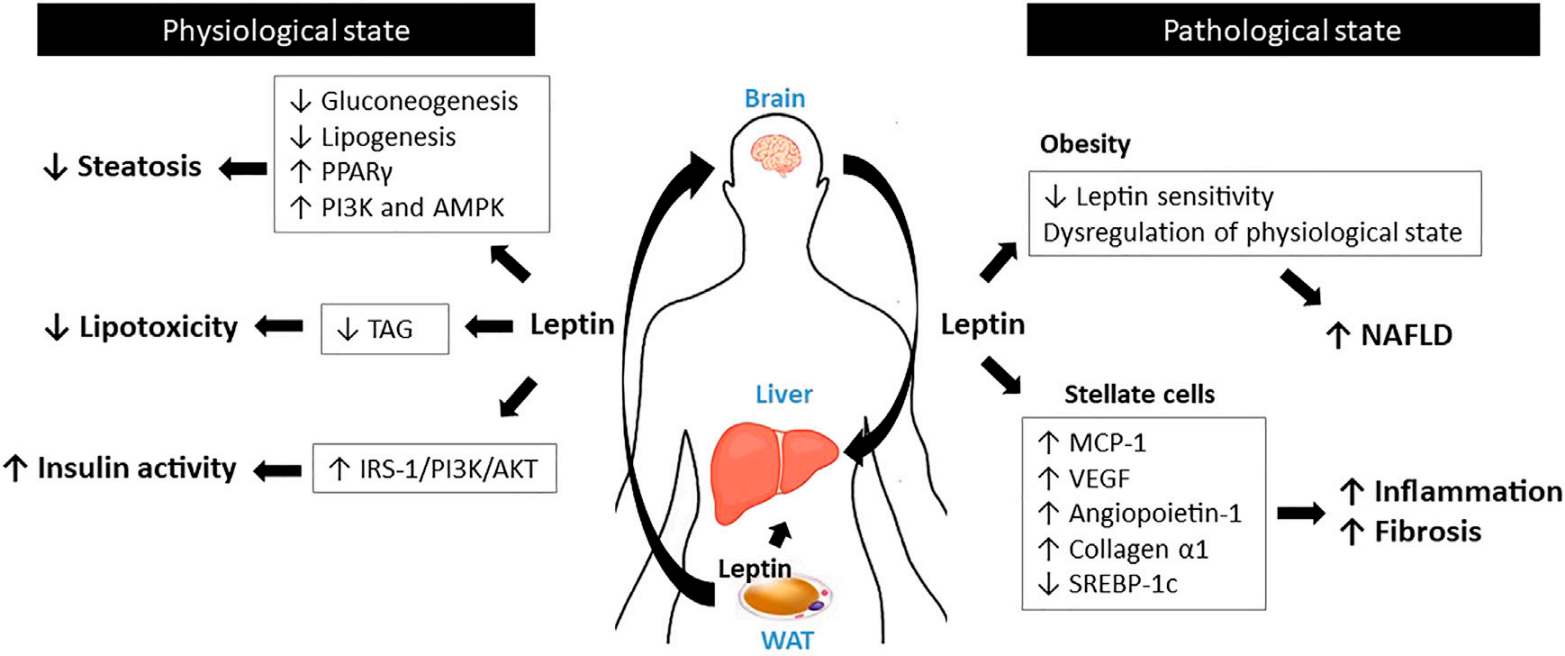
Schematic diagram for the roles of leptin in the liver.

Despite these positive reports on the effect of leptin on the liver, the efficacy of leptin in preventing liver steatosis seems limited by obesity. This is due to leptin resistance, one of the major features observed in obese individuals, where circulating leptin levels are extremely high compared to those in lean individuals (Izquierdo et al., 2019). Despite high concentrations, leptin resistance leads to failure of leptin action, which inhibits the modulation of hepatic glucose metabolism and insulin response (Engin, 2017; Rizwan et al., 2017). Increased fat mass in obesity causes chronic inflammation and increases the expression of numerous adipokines, including leptin. The increased leptin levels in obese status appear to boost hepatic pro-inflammatory and pro-fibrogenic responses, thus damaging the liver (Saxena and Anania, 2015). Indeed, *ob/ob* mice and *fa/fa* Zucker rats fail to develop fibrosis during hepatic steatosis or toxin administration (Leclercq et al., 2002; Ikejima et al., 2005), demonstrating that leptin plays a significant role in this process. The pro-fibrogenic action of leptin involves hepatic stellate cells (HSCs), which are liver-specific pericytes (Tsuchida and Friedman, 2017). Once activated, but not quiescent, HSCs express leptin (Potter et al., 1998). Leptin supports the proliferation and survival of HSCs via ERK- and Akt-dependent phosphorylation pathways (Saxena et al., 2004). In addition, leptin in HSCs stimulates the expression of pro-inflammatory and angiogenic cytokines, such as monocyte chemoattractant protein-1 (MCP-1), VEGF, angiopoietin-1, and collagen α1, which results in higher hepatic collagen expression and inflammation (Aleffi et al., 2005; Yan et al., 2012). Leptin also inhibits the expression of sterol regulatory element-binding protein 1c (SREBP-1c), an inhibitor of fibrogenesis, via the β-catenin pathway in isolated HSCs (Zhai et al., 2013). Despite this evidence in experimental *in vitro* and *in vivo* models, conflicting data have been obtained in human patients with NAFLD, regarding the role of leptin in hepatic inflammation and fibrosis (Polyzos et al., 2015); this has been a major obstacle in testing leptin therapy for this disease. However, leptin therapy has been actively used in patients with lipodystrophy, a disorder characterized by fat loss, severe insulin resistance, and NAFLD and steatohepatitis (NASH) (Akinci et al., 2018). Notably, patients with lipodystrophy display low circulating levels of leptin (hypoleptinemia) (Chong et al., 2010). In 2002, leptin replacement was initially tested in nine female patients with lipodystrophy, and the therapy was shown to be effective in treating the disease (Oral et al., 2002). Specifically, leptin therapy improves glycemic control and decreases triglyceride levels, thus effectively improving the symptoms of NASH (Polyzos et al., 2019; Baykal et al., 2020) (Figure 3).

## Pancreas

Leptin controls glucose homeostasis mainly via its actions on the hypothalamus (D'souza et al., 2017). Pancreatic β-cells are another important target of leptin to regulate glucose metabolism (Marroquí et al., 2012). The functional receptor LepRb is detected in various β-cell lines, including MIN-6, βTC-3, and INS-1 (Kieffer et al., 1996; Tanizawa et al., 1997; Ahren et al., 1999), as well as in the islets of rodents and humans (Emilsson et al., 1997; Poitout et al., 1998; Seufert et al., 1999b). On the contrary, LepR expression is not detected in glucagon-expressing α-cells; thus, leptin action seems limited to β-cells (Soedling et al., 2015). Under steady-state conditions, leptin signaling in the CNS seems to govern pancreas physiology, rather than its direct action in pancreas.

In physiological states, leptin inhibits insulin gene expression in β-cells (Zhao et al., 2020). Insulin inhibition by leptin is mediated by the activation of JAK/STAT3 and STAT5b signaling (Morton and Schwartz, 2011). In INS-1 cells and in human pancreatic islets, Laubner et al. demonstrated that activation of STAT3 and STAT5b induces SOCS3 expression, which in turn suppresses preproinsulin one gene promoter activity (Laubner et al., 2005). This notion was further supported by (Pedroso et al., 2014)who showed that inactivation of SOCS3 in LepR-expressing cells protects mice against diet-induced insulin resistance, indicating that SOCS3 is a critical downstream signaling mediator of leptin to orchestrate glycemic control (Pedroso et al., 2014). In addition to gene expression, leptin inhibits insulin secretion from β-cells by modulating multiple steps of the hormone secretion mechanism (Kulkarni et al., 1997; Kuehnen et al., 2011). Leptin directly inhibits glucose transport into β-cells by inhibiting GLUT2 phosphorylation (Lam et al., 2004). Leptin also inhibits the activation of ATP-dependent potassium channels and the subsequent reduction of Ca^2+^ influx (Kieffer et al., 1997; Seufert et al., 1999a), which interrupts the exocytosis of insulin from β-cells.

Leptin has been shown to contribute to maintaining β-cell mass by modulating the proliferation and apoptosis of β-cells. LepR-deficient *fa/fa* Zucker rats and *db/db* mice develop reduced β-cell mass, which is associated with aging (Lee et al., 1994; Dalbøge et al., 2013), although leptin-deficient *ob/ob* mice exhibit normal islet mass (Bock et al., 2003). In mice, pancreas-specific LepR knockout directly affects β-cell growth and function (Morioka et al., 2007). Leptin treatment enhances cell proliferation in many β-cell lines such as RINm5F and MIN-6, as well as in rat fetal islet cells (Islam et al., 1997; Tanabe et al., 1997; Islam et al., 2000). These proliferative effects of leptin are mediated by activation of the MAPK pathway and c-fos, which are critical regulators of the cell cycle (Zhang and Liu, 2002). In addition, though the results are conflicting, leptin influences β-cell apoptosis. Some studies suggest that leptin suppresses apoptotic β-cell death. In rat islets, leptin increases intracellular fatty acid oxidation, and subsequently depletes triglyceride accumulation in islets, thereby preventing lipotoxicity-induced apoptosis (Shimabukuro et al., 1997).

Mechanistically, *fa/fa* Zucker rats develop reduced β-cell mass due to increased lipotoxicity (Pick et al., 1998; Wang et al., 1998). These results suggest that β-cell lipotoxicity may be a key mediator linking leptin resistance and the development of diabetes (Unger and Roth, 2015; Ercin et al., 2018). In addition, leptin reduces cellular nitric oxide levels by suppressing inducible NO synthase (iNOS) expression (Okuya et al., 2001) and increasing anti-apoptotic Bcl-2 expression in rat pancreatic islets (Shimabukuro et al., 1998), thereby exerting anti-apoptotic effects. However, other studies have shown that leptin enhances β-cell apoptosis. In human islets and INS 832/13 cells, long-term exposure to leptin triggers β-cell apoptosis through activation of the JNK pathway (Maedler et al., 2008). Similarly, chronic exposure of human islets to leptin leads to impaired β-cell function, caspase-3 activation, and apoptosis due to increased IL-1β expression and reduced IL-1 receptor antagonist expression (Maedler et al., 2004). This discrepancy between the anti- and pro-apoptotic effects of leptin may be due to different experimental designs. Indeed, most pro-apoptotic effects were observed when the cells were chronically treated with leptin (Maedler et al., 2004; Maedler et al., 2008), whereas an anti-apoptotic effect was observed with short-term treatment (Okuya et al., 2001).

**FIGURE 4:**
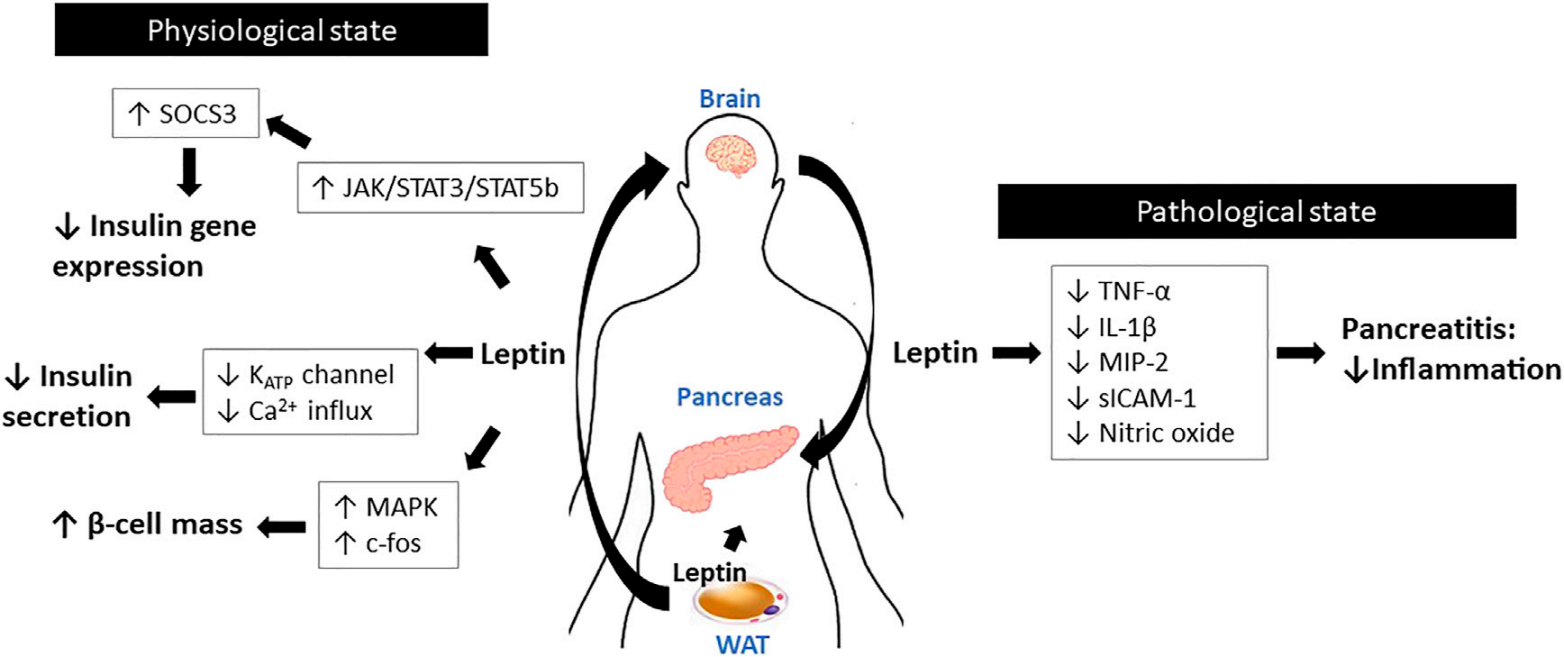
Schematic diagram for the roles of leptin in the pancreas.

Several *in vivo* studies have reported that leptin may exert a protective effect on pancreatitis. When acute pancreatitis is induced, *ob/ob* mice exhibit higher pathologic scores and intestinal permeability relative to wild-type mice, indicating that absence of leptin signaling aggravates intestinal inflammation (Ye et al., 2018). In rats with caerulein-induced pancreatitis, leptin administration (1 or 10 μg/kg BW, i. p.) significantly reduced the weight of the pancreas, histological manifestations of inflammation, and the expression of TNF-α and iNOS (Konturek et al., 2002). In the same pancreatitis model, leptin administration (10 μg/kg BW, i. p.) attenuated disease severity, which is mediated by reduced levels of proinflammatory cytokines, such as MIP-2, TNF-α, IL-1β, and sICAM-1, and nitric oxide (Gultekin et al., 2007). In the clinical setting, higher plasma leptin concentrations are associated with acute pancreatitis; thus, leptin may be a possible predictor of disease severity (Konturek et al., 2002; Kerem et al., 2007). Leptin is also associated with persistent hyperglycemia, as shown in the early course of acute pancreatitis (Kennedy et al., 2016). However, no clinical trials have examined exogenous leptin treatment in patients with acute pancreatitis (Figure 4).

## Discussion

In this section, we discuss the roles of leptin in the digestive system, including the stomach, intestine, liver, and pancreas. The current knowledge indicates that leptin plays critical but complex roles in these tissues, where its action appears to differ in the physiological and pathological states (Figure 1- 4). The functional leptin/LepR axis is required for maintaining normal energy homeostasis and systemic glucose homeostasis. In addition, leptin exerts protective effects by supporting cell proliferation, improving tissue repair, and preventing non-adipocyte lipotoxicity. However, leptin action is not always protective, particularly during pathologic conditions. Increased leptin and LepR levels are often observed in several inflammation-related diseases, where activated leptin signaling accelerates inflammatory response by promoting T-helper cell responses and supporting the production of proinflammatory cytokines (Fernández-Riejos et al., 2010). Of note, relatively little has been investigated a direct action of leptin in the digestive system. Indeed, most of the studies have examined the effect of central or peripheral leptin administration, which activates leptin signaling in the central nervous system. In that most of the leptin functions have been attributed to its action in the brain, there is no strong evidence to demonstrate leptin acts directly on peripheral tissues by binding receptors expressed in these tissues. Further studies using tissue-specific knockout models to limit the receptor expression are highly warranted.

Leptin replacement therapy is enormously beneficial in individuals with congenital leptin deficiency, which restores their energy homeostasis, neuroendocrine system, and glucose metabolism (Perakakis et al., 2021). As mentioned above, leptin administration is also widely used in patients with lipodystrophy. Since lipodystrophy is a medical condition characterized by degenerative adipose tissue, leptin production in patients is significantly reduced (Grewal et al., 2020). Therefore, administration of human recombinant leptin improves many metabolic defects in patients with lipodystrophy (Petersen et al., 2002). Since its discovery, leptin has been considered an attractive therapeutic target for the treatment of obesity and type 2 diabetes, due to its potency for the endocrine control of energy balance (Friedman, 2019). However, unlike that in congenital leptin deficiency, leptin concentrations are elevated in obesity owing to large fat mass, and systemic leptin signaling is blunted despite high circulating leptin levels. This status has been defined as “leptin resistance”, which indicates poor responsiveness to leptin (Gruzdeva et al., 2019). Therefore, exogenous leptin treatment results in no or minimal effects on body weight and neuroendocrine function (Heymsfield et al., 1999; Mantzoros and Flier, 2000). Similarly, leptin therapy only produces modest effects on insulin sensitivity in type 2 diabetes due to leptin resistance (Mittendorfer et al., 2011).

Several *in vivo* experiments have explored the possible use of recombinant leptin or leptin antagonists in digestive organs. As discussed above, leptin augments proinflammatory responses and enhances susceptibility to autoimmune diseases, including UC (La Cava et al., 2004), which implies that neutralizing leptin action using a leptin antagonist may ameliorate the disease symptoms. Indeed, Singh et al. examined a leptin antagonist (PEG-MLA) to treat induced colitis in IL-10 knockout mice (Singh et al., 2013). Interestingly, they reported that PEG-MLA administration reduces systemic and mucosal inflammatory cytokine expression, thereby significantly attenuating the overall clinical features of colitis-associated pathogenesis. However, treatment with leptin or leptin antagonists has not been actively examined in clinical settings, mainly because the underlying mechanisms of leptin action in the digestive system are still unclear. Furthermore, leptin action is multifunctional and complex, which modulates numerous signaling pathways thus increasing the likelihood of an adverse reaction. For example, administration of a leptin antagonist can result in inhibition of the JAK/STAT pathway, a major pathway involved in cell division, cell death, and immunity, which may cause unexpected side effects. In addition, LepR expression is widespread in the body, which makes it difficult to confine the treated leptin/leptin antagonist to the target site. For instance, an excessively high dose of a leptin/leptin antagonist may Singh et al., 2013 cause serious deleterious effects on neuroendocrine function, as the hypothalamus is the most sensitive tissue in response to leptin. Therefore, more information is needed regarding the molecular mechanisms of leptin in the digestive system to consider manipulation of leptin signaling in these tissues as a novel therapeutic approach.
